# World health order

**DOI:** 10.7189/jogh.14.03023

**Published:** 2024-04-05

**Authors:** Carlos Javier Regazzoni

**Affiliations:** 1 Institute of Global Health, Faculty of Health Sciences, Kennedy University, Buenos Aires, Argentina; 2 Committee on Global Health and Human Security, Argentine Council on Foreign Relations, Buenos Aires, Argentina

The efforts to promote public health operate in a world heavily influenced by politics, power struggles, and conflicting business interests, often leading to adverse outcomes. Critical issues such as access to pharmaceuticals, vaccine distribution, health care system performance, and international organisations’ priorities on climate change or drug patents stand as contentious arenas with direct implications for human lives. Through the transfer of risk or the priorities setting of business [1], the current world economic and political order creates global forces pressing on population health. This contradictory context gives rise to what may be perceived as a ‘world health order’, a byproduct of the broader world order that perpetually emerges from the shadows of global power struggles. It would be naive to attempt to reshape the former without understanding the latter. Conversely, to achieve a more equitable world order, it is imperative to prioritise and reestablish health equity as a central principle of the global agenda. In this context, global health studies should seek to deepen their engagement with a geopolitical concept that comprehensively addresses the power dynamics and geostrategic factors influencing the population’s health; the framework of the world health order could fulfil this commitment.

Global health is focused on the vital nexus of societal, economic, and political determinants that are instrumental in shaping world health outcomes. This discipline, then, provides the quintessential platform for rigorous debate on the impact of business, power, and geopolitical interests on public health [2]. The exploration of any interplay between world order, as defined by international affairs scholars and world health, directly falls within its domain.

Large powers repeatedly overshadow health priorities, often placing geopolitical and economic strategic interests above health-centric approaches to public interest and health issues. Healthcare providers and policymakers in the field of public health must acknowledge the significance of a large power agenda on health policies and systems across global and national domains. It has been stressed that stronger bonds between global health research and the body of political literature are needed [3]. To achieve this ambitious goal, it is imperative to develop a more comprehensive understanding of world order and its implications on health outcomes [4].

We believe that a notion of a ‘world health order’ would offer a thorough framework to examine and comprehend the intricate interplay between geopolitics and the present levels of population health and equality. Establishing and enhancing this ‘shared language’ with geopolitics and international economics is decisive, as it is pivotal for precisely deciphering global dynamics and formulating more efficient health policies. The concept of a world health order could reduce uncertainty in the channels of communication between powerful international actors and the health priorities proposed by medical science and other health-equality-motivated transnational actors.

## DEFINING WORLD ORDER

Albeit disagreements among scholars, ‘world order’ would be defined as a group of state and non-state transnational agents, conscious of certain common interests and values, that recognise themselves as bound by a shared set of rules in their interactions and collectively contribute to the functioning of common institutions. Politically speaking, the world integrates a set of commonly accepted rules that define the limits of permissible actions for transnational actors, along with a balance of power that ensures restraint among these same agents when confronting these written and consuetudinary norms, constituting in this way an order [5].

World order, then, signifies the current structure and dynamics of the international system, the balance of power among nations, the guiding principles and norms of statecraft, and the underlying mechanisms that enable diplomatic cooperation and management of conflicts [6]. The nonexistence of such a theoretical foundation would render the understanding and involvement with the global system impracticable.

The concept of power is inherently linked to the very notion of world order. Consequently, any hypothetical effect of the world order on population health ought to be assumed as the direct outcome of power-driven decision-making at a global scale. This dynamic of power shapes world order, the eventual main driver of global health outcomes, progress and disparities. Prominent global health reports explicitly target the role of power and politics operating at various levels to sustain health inequities [3]. Numerous examples illustrate how major power dynamics have influenced public health decisions and outcomes. For instance, the underrepresentation of intended beneficiaries in global health decision-making, particularly with organisations’ headquarters predominantly in high-income countries, underscores international power dynamics impacting global health policymaking [7]. Moreover, from 1998–99, the conflict between President Mandela’s government and United States (US) pharmaceutical firms escalated globally. South Africa’s efforts to lower drug prices through legislation prompted immediate US pressure to block generic drug imports and compulsory licensing. This battle highlighted conflicts over patent laws, global intellectual property rules, and power imbalances between developing countries and the pharmaceutical industry [8]. More recently, the diplomatic stand-off between the USA and China blocked agreements at the World Health Organization (WHO), the United Nations (UN) Security Council, the Group of Twenty (G20), and the Group of Seven (G7) during the coronavirus disease 2019 (COVID-19) pandemic. Due to strategic reasons, the confrontation between both superpowers had indubitable consequences on global health in a period of crisis [9].

Nevertheless, despite the recognition of power’s influence on global health initiatives and outcomes, the utilisation of social scientific theories and methodologies to examine power is hardly practised within the realm of health policy research and theorisation. A precise world health order framework would solve this gap.

## A PROPOSED DEFINITION FOR WORLD HEALTH ORDER

To our knowledge, a universally accepted definition of ‘world health order’ remains elusive. While the idea of clear interrelations between world powers and health outcomes is alluded to and empirically substantiated in numerous documents and scholarly papers, a precise and comprehensive definition of the world health order is lacking. We argue that establishing a working definition of the world health order is crucial for comprehensively understanding the myriad factors that ultimately influence the health status of populations worldwide. For global health initiatives to successfully promote health equity worldwide, they must effectively transform what will be called the ‘world health order’.

To begin with, we discuss a type of social order [10]. Such an order is a hierarchically structured framework in which social agents’ roles, responsibilities, and behaviours are defined and regulated by established group-sanctioned norms, goals, and rules. This configuration ensures predictable outcomes by aligning agents’ actions with set expectations. This predictability is maintained through power balances and the enforcement of rules.

In parallel with the characterisation of ‘world order’, ‘world health order’ could be defined as the structure and dynamics of the international system as they influence health outcomes. It encompasses the balance of power among such health-influencing global actors, the guiding principles and norms governing global health, and the mechanisms fostering cooperation, managing health inequalities, and advancing population health.

World health order exists only if health issues gain prominence in politics, power, and business. In fact, health care expenditure accounts for 10% of the global gross domestic product [11], evidencing a sizable economic impact. Furthermore, sectors intimately linked to global health outcomes, such as the food and tobacco industries, also control a considerable portion of the world economy. There is evidence of how the Commercial Determinants of Health undermine health equity and can threaten progress across health-related goals [12]. Likewise, the allocation of enormous resources to health care exercises considerable influence over the wider economy and financial sector, thereby attracting the interest of banks and financial institutions [13] with their own agendas for the system. These powerful entities demonstrate a particular concern for the influence of health care expenditure choices on fiscal balance and labour expenses, thereby highlighting the crucial intersection between health care financing and economic stability. Moreover, health issues are subject to extensive regulation and policymaking across the globe, often adhering to international standards and under the supervision of international agencies; this regulatory landscape contributes to establishing a form of health governance, shaping the global health setting. Consequently, the global health landscape is politically and economically extended enough to be shaped by the strategies and agendas of key world players, each driven by its own sometimes divergent interest, employing unique ways of lobbying and persuasion, and finally exerting considerable influence on world health [14]. Central to this dynamic is the reality that these forces do not necessarily stem from health priorities, evidence-based recommendations, or values of equality and transparency. Instead, they result from the same power dynamics that determine the previously analysed world order.

Global health outcomes are decisively shaped by global players’ powerful influences and interests, alongside public health challenges such as climate change, pandemics, international risk transfer, and the necessity of ensuring global access to the best medical practices for all. In this regard, isolated national health systems face nearly insurmountable challenges in addressing their populations' health care needs alone [1]. Consequently, it is precisely from the interplay of these national health systems with global actors and global public health challenges that the world health order emerges. This order dictates health priorities, resource allocation, health care access, and equitable distribution of services and technologies. It is a dynamic construct responsive to global trends, scientific advancements, and geopolitical changes.

## ADVANCING ON PREVIOUS THEORIES

The world health order framework introduces a robust geopolitical dimension to global health, integrating aspects traditionally reserved for international affairs and its notion of world order. Concepts and theories from international relations have been previously applied to better understand the role of power in shaping positions, negotiations, and outcomes under the perspective of global health diplomacy [15]. The distinction lies in the practical application of health diplomacy vs the theoretical utility of the world health order framework. The latter serves as a critical lens to grasp a complex and decisive reality wherein international actors prioritise their power agendas, even at the expense of the population’s health. This framework demands that such agendas be analysed in strategic terms, akin to geopolitical evaluation, to understand how power dynamics influence global health priorities and outcomes.

Potential criticisms of the existence of a world health order could sustain that commitment to global health exceeds geopolitical interests and world business agendas; this is a notion systematically refuted by every single piece of evidence [16] and starkly highlighted during the pandemic. Altruism seems far away from global health issues, so it is necessary to include strategic interests in its analysis. Enriching global health analysis with the realpolitik perspective that lies beneath world order drivers will give a sense of realism to every altruistic health endeavour.

## POLICY IMPLICATIONS

World order is shaped by the interplay of great powers and transnational corporations, establishing asymmetric power negotiations where public health is seldom in a balanced position. Health agenda has been difficult to advance in trade agreements relating to food, tobacco, and drugs access. The term ‘realpolitik’ has assertively been used to describe this phenomenon [17]. The most striking feature of international negotiation tables where the interests of global public health are potentially affected, besides the asymmetry of power, is the appeal to different sources of authority, which varies according to the actor under consideration. In a study [18], researchers identify four types of authority mobilisation: institutional authority, legal authority based on an international legal framework, networked authority through cross-referencing between actors, and expert authority through evidence. These bases of authority were claimed differently by actors from businesses, politics, or public health sectors. These types of studies are necessary to improve the performance of global health and health diplomacy initiatives. The world health order concept will help to increase public health practitioners’ awareness about real strategic interests when populations health could be impacted.

## CONCLUDING REMARKS

We adhere to the idea that global health addresses medical and health issues with worldwide influence and that its primary aim is to find international solutions to these concerns, harnessing academic research and scientific studies to promote overall health, increase equity, and reduce disparities [3]. Assuming that each sovereign State and large corporation tends to look primarily and foremost at its own separate and several interests, a better understanding of this power-driven dynamic is mandatory. For instance, the accessibility and progress in pharmaceuticals indispensable for human existence often carry significant geopolitical resonances that can sometimes conflict with poorer nations’ interests [19]. Thus, their interweaving with geopolitical dynamics warrants serious attention, in the conviction that ‘no invisible hand is at work to sort out the geopolitical marketplace’ [20].

In this context, the world health order likely emerges as the primary focus for global health studies and initiatives. Thus, the main question for global health scholars and practitioners should be what form of world health order would most effectively enhance health equity and improve health outcomes globally [21]. Then, we should focus on world order in geopolitical terms and work on the real drivers of change. The strategic interests of major powers, large corporations, and strong international agencies relentlessly defy the advancement of the population’s health agenda. In this context, more studies on realpolitik interests and, finally, modelling the public health agenda should be encouraged; methodology requirements should be broadened beyond the traditional biomedical research frameworks. Moreover, open discussions regarding power interests, methods, and drivers, should be included in health forums to foster a more comprehensive approach to what ultimately constitutes a world health order shaped by greed and power goals. A deeper understanding of this dynamic will significantly benefit the public interest, which health care teams advocate for.
